# Transcriptional Responses of Creeping Bentgrass to 2,3-Butanediol, a Bacterial Volatile Compound (BVC) Analogue

**DOI:** 10.3390/molecules22081318

**Published:** 2017-08-16

**Authors:** Yi Shi, Kuiju Niu, Bingru Huang, Wenhui Liu, Huiling Ma

**Affiliations:** 1 College of Grassland Science, Gansu Agricultural University, Lanzhou 730070, China; shiyi214@126.com (Y.S.); niukuiju@163.com (K.N.); 2 Key Laboratory of Grassland Ecosystems, The Ministry of Education of China, Lanzhou 730070, China; 3 Department of Plant Biology and Pathology, Rutgers University, New Brunswick, NJ 08901, USA; huang@aesop.rutgers.edu; 4 Key Laboratory of Superior Forage Germplasm in the Qinghai-Tibetan Plateau, Qinghai Academy of Animal Science and Veterinary Medicine, Xining 810016, China; qhliuwenhui@163.com

**Keywords:** bacterial volatile compounds, 2,3-butanediol, creeping bentgrass, turfgrass transcriptome, induced disease resistance, RNA-seq

## Abstract

Bacterial volatile compounds (BVCs) have been reported to enhance plant growth and elicit plant defenses against fungal infection and insect damage. The objective of this study was to determine transcriptomic changes in response to synthetic BVC that could be associated with plant resistance to *Rhizoctonia solani* in creeping bentgrass. The 2,3-butanediol (BD) (250 µM) was sprayed on creeping bentgrass leaves grown in jam jars. The result showed that synthetic BD induced plant defense against *R. solani* for creeping bentgrass. Transcriptomic analysis demonstrated that more genes were repressed by BD while less showed up-regulation. BD suppressed the expression of some regular stress-related genes in creeping bentgrass, such as pheromone activity, calcium channel activity, photosystem II oxygen evolving complex, and hydrolase activity, while up-regulated defense related transcription factors (TFs), such as basic helix-loop-helix (bHLH) TFs, cysteine2-cysteine2-contans-like (C2C2-CO) and no apical meristem TFs (NAC). Other genes related to disease resistance, such as jasmonic acid (JA) signaling, leucine rich repeats (LRR)-transmembrane protein kinase, *pathogen-related (PR)* gene 5 receptor kinase and nucleotide binding site-leucine rich repeats (NBS-LRR) domain containing plant resistance gene (*R*-gene) were also significantly up-regulated. These results suggest that BD may induce changes to the plant transcriptome in induced systemic resistance (ISR) pathways.

## 1. Introduction

Plant growth-promoting rhizobacteria (PGPR) are a wide range of root-colonizing bacteria with the capacity to enhance plant growth under normal and stress conditions [1,2,3]. Application of some PGPR strains to seeds or seedlings has been found to enhance plant growth and induce disease resistance [4,5,6,7]. The PGPR promote disease resistance in many plant species, such as bean (*Glycine max*) [8], carnation (*Dianthus caryophyllus*) [9], cucumber (*Cucumis sativus*) [10], radish (*Raphanus sativus*) [11], tobacco (*Nicotiana tabacum*) [12], tomato (*Lycopersicon esculentum*) [13] and thale cress (*Arabidopsis thaliana)* [14]. The positive effects of some PGPR on plant growth may be due to the effects of BVCs, serving as an elicitor to induce plant defense systems against diseases [15].

There are a number of studies demonstrating plant resistance to pathogens under different conditions [16,17]. The major forms of plant-induced resistance are induced systemic resistance (ISR) and systemic acquired resistance (SAR). ISR was mostly reported related with the jasmonate/ethylene (JA/ET)-dependent pathway [18], while SAR was known to be promoted with salicylic acid and NPR1 induction [19], which was dependent on the expression of plant pathogen-related genes. PGPR- and BVC-induced plant disease resistance was mostly reported signaled as an ISR form, which transport through the jasmonate/ethylene (JA/ET)-dependent pathway. However, the mechanism of BVC-induced plant disease resistance was still unclear. The induced resistance in *Arabidopsis* triggered by a volatile compound released from *Bacillus subtilis* GB03 induced disease resistance in *Arabidopsis*, which was independent of salicylic acid, NPR1, and JA signaling pathways [20]. *Arabidopsis* treated by *Bacillus amyloliquefaciens* IN937a also showed induced systemic resistance, while the pathway is independent of all signaling pathways that were tested, which opens up the possibility that additional BVCs utilize alternative pathways *in planta* to trigger disease resistance [20]. A recent proteomics study that employed *Arabidopsis* tissue exposed to GB03 revealed that five representative ET biosynthesis-related genes were significantly up-regulated by this treatment [21]. In wheat (*Triticum aestivum*), a gene encoding a subtilisin-like protein was up-regulated after seed treatment with *Pseudomonas aeruginosa* [22], and the plant showed resistance to *Blumeria graminis f*. sp. *tritici* or *Chochliobolus sativus*. Root inoculation of maize (*Zea may*s) with *Trichoderma virens* induced genes encoding an allene oxide synthase (ZmAOS), hydroperoxide lyase (ZmHPL) and 12-oxophytodienoic acid reductase (ZmOPR-7), which contributes to the induced resistance of maize to *Colletotrichum graminicola* [23]. The application of synthetic BD and a volatile extract collected from PGPR had similar effects on disease protection, which is comparable to that induced by direct PGPR inoculation [20]. The compound 2*R*,3*R*-BD, which is one of the racemic forms of BD, can also induce resistance in tobacco against *Erwinia carotovora* subsp. *carotovora* SCC1 [24]. Soil drenching of 2r, 3r-BD reduced disease infection in *Agrostis stolonifera* by 20–40% for the fungal pathogens, *Microdochium nivale*, *Rhizoctonia solani* or *Sclerotinia homoeocarpa* compared to the water control [25]. Despite the knowledge of the positive effects of BD, the underlying mechanisms are not well understood.

Synthetic fungicides are commonly and frequently applied to turfgrass to provide disease control, but the extensive and heavy application of fungicides can be costly and also potential pollutants to the environment [26]. Using non-fungicidal compounds to induce plant natural defense for disease resistance is a sustainable approach to maintain high quality turfgrass. In order to better understand the mode of action of BVCs, it is important to identify genes responsive to BVC that may control induced disease resistance in plants. The objective of this study was to determine transcriptomic changes in response to synthetic BVC, BD, that could be associated with plant resistance to *R. solani* in creeping bentgrass (*Agrostis stolonifera*). Creeping bentgrass, a widely used turfgrass in temperate climatic regions, is very susceptible to *R. solani* [27]. The knowledge of the molecular factors involved in BVC-activation of plant defense systems will provide further insights into mechanisms of induced systemic resistance in plants and promote the use of natural or synthetic BVCs or PGPR as alternatives to fungicides for disease control.

## 2. Results

### 2.1. Disease Assessment of Creeping Bentgrass Treated with BD and the Antimicrobial Activity of BD

**Figure 1:**
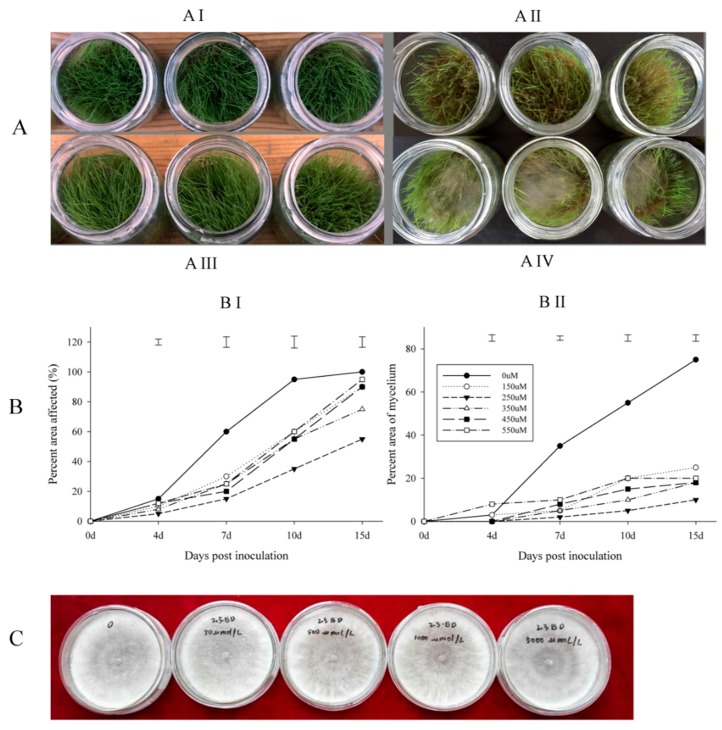
Butanediol (BD)-reduced *R. solani* caused damage to creeping bentgrass and suppressed mycelium growth on grass, while no growth inhibition of *R. solani* showed on BD-containing PDA plate. A-BD effect on grass (**AI**) 250 µM BD-treated grass without inoculation; (**AII**) 250 µM BD-treated grass with inoculation; (**AIII**) Water-treated grass without inoculation; (**AIV**) Water-treated grass with inoculation. (**B**) BD effect by different concentrations on affected leaf area and mycelium growth (**BI**) BD effect on affected leaf area; (**BII**) BD effect on mycelium growth; Bars on the top of the figure means the least significant difference between each concentration; (**C**) BD effect on fungus growth on PDA plate.

The time of symptom appearance did not differ between plants treated with water or BD (Figure 1A). The disease levels of plants treated with different concentrations of BD were not significantly different at four days post inoculation. At seven and 10 days post inoculation, BD-treated grass showed significantly fewer disease symptoms in terms of affected area. The treatment with 250 µM BD showed greater suppression of disease symptom development than the other concentrations compared with the control (Figure 1B). This is why 250 µM BD was used for RNA sequence (RNA-seq) sample preparation.

An examination of the direct antifungal activity of butanediol (BD) showed no visible effects on fungal growth or morphology when *R. solani* was grown on PDA plate amended with 0, 50, 100, 500, 1000, or 5000 µM BD (Figure 1C).

### 2.2. Transcriptome Sequencing and Read Statistics

**Figure 2:**
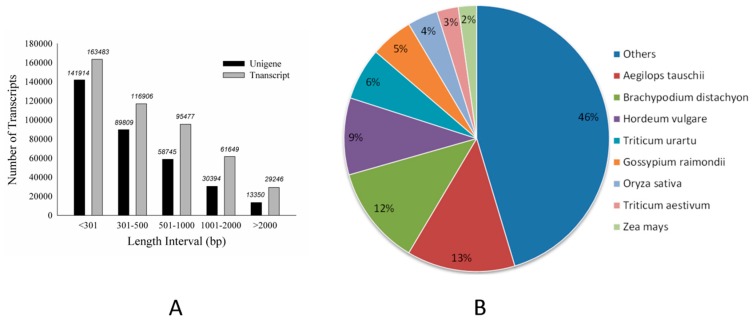
The length distribution of de novo assembly and taxonomic source of BLAST matches for creeping bentgrass unigenes. (**A**): The length distribution of de novo assembly. (**B**): Taxonomic source of BLAST matches.

**Figure 3:**
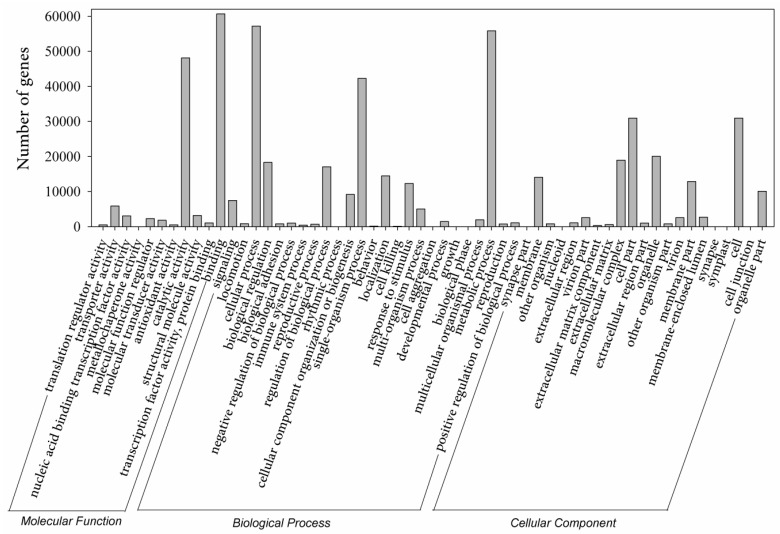
Gene ontology (GO) distributions for the creeping bentgrass transcriptome. The main functional categories in the biological process, cellular component and molecular functions found in the transcriptome. The ordinate indicates the number of unigenes. Bars represent the numbers of assignments of creeping bentgrass proteins with BLASTx matches to each GO term. One unigene may be matched to multiple GO terms.

Using Illumina-based 2 × 150 bp paired-end reads sequencing, a total of 258,606,402 reads were produced for the libraries of 0, 1, 4 and 7-day BD-treated samples, which generated 25 Gb of data. Cleaned reads were de novo assembled and produced a set of 466,761 transcripts using Trinity. The summary of sequencing and assembly can be found in Table 1. The length distribution of the assembled transcripts are shown in Figure 2A. We selected 334,212 sequences (71.60% of the total transcripts) as unigenes to query against several commonly used databases for function annotation. The general blast result is exhibited in Table 1. Further investigation showed that among the result of Basic Local Alignment Search Tool (BLAST) with non-redundant database (NR), creeping bentgrass unigenes were significantly similar to *Aegilops tauschii* proteins (17,166, 13%), followed by *Brachypodium distachyon* (15,384, 12%) and *Hordeum vulgare* (12,167, 9%) (Figure 2B). The other unigenes (26,127, 20%) were similar to the proteins of important gramineous plants, including *Triticum urartu*, *Oryza sativa*, *Zea mays* and *Sorghum bicolor*. The distribution of protein assignments to specialized Gene Ontology (GO) terms indicates that creeping bentgrass sequences represent proteins from a diverse range of functional classes (Figure 3).

**Figure 4:**
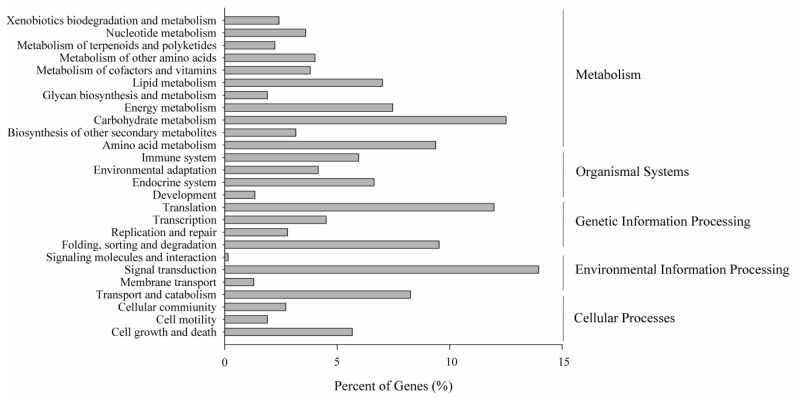
KEGG classification of the creeping bentgrass transcriptome. Unigenes involved in the metabolism pathways by KEGG classification are divided into five groups as showed on the right side of the figure.

To correlate creeping bentgrass unigenes with known metabolic pathways, the KEGG Automatic Annotation Server (KAAS) was used to assign sequences with Kyoto Encyclopedia of Genes and Genomes (KEGG) orthology (KO) terms and their respective KEGG maps [28,29]. A total of 41,963 (12.56%) assembled unigenes were associated with 277 pathways, which can be classified into a 26-pathway hierarchy 2 (Figure 4) related with plant life.

### 2.3. Differentially Expressed Genes under the Treatment of BD

**Figure 5:**
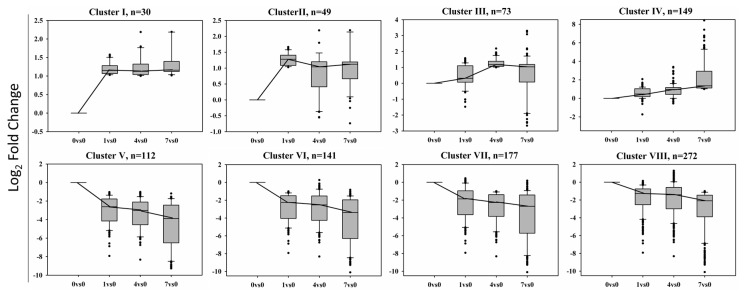
Clustering of differentially expressed genes in the transcriptome of creeping bentgrass leaf tissue treated with BD. N is the number of transcripts found in each cluster. Cluster I and V contain BD-induced or -repressed genes at all three time points, respectively; Cluster II, III and IV contain BD-induced genes at 1, 4 and 7 DPT, respectively; Cluster VI, VII and VIII contain BD-repressed genes at1, 4 and 7 DPT, respectively. A number of differentially expressed genes were included in more than one cluster according to their expression profiles. The y-axis represents the log_2_(foldchange) of expression value. |log_2_(foldchange)| > 1 (*q*-value < 0.005) was designated as differentially expressed genes.

Using the DEGseq Bioconductor package, the differentially expressed genes were filtered by the log_2_(foldchange) of 1 and the *p*-value adjusted (*q*-value) < 0.005 [30] to avoid false positives [31]. Based on these values, the unigenes were determined to be unique or shared with the compared combinations (DPT 1 vs. DPT 0, DPT 4 vs. DPT 0, DPT 7 vs. DPT 0), as shown in Figure 5.

The differentially expressed genes (DEGs) were grouped according to their expression patterns across four libraries into eight clusters (Figure 5). Cluster I contains 30 transcripts up-regulated with BD treatment, they were shared by 1 DPT, 4 DPT and 7 DPT compared with 0 DPT. Cluster V has 112 transcripts down-regulated with BD treatment and shared by 1 DPT, 4 DPT and 7 DPT compared with 0 DPT. Cluster II is composed of 49 transcripts that are abundant at DPT 1 VS. DPT 0, Cluster III is composed of 73 transcripts that are abundant at DPT 4 VS. DPT 0 and Cluster IV is composed of 149 transcripts that are abundant at DPT 7 VS. DPT 0. Conversely, Cluster VI is composed of 141 transcripts that are down-regulated at 1 DPT compared with 0 DPT, Cluster VII is composed of 177 transcripts that are down-regulated at 4 DPT compared with 0 DPT and Cluster VIII is composed of 272 transcripts that are down-regulated at 7 DPT compared with 0 DPT. A number of differentially expressed genes were included in more than one cluster according to their expression profiles. Although there are more than 1000 genes presented in the Figure 5, there are only 504 genes in total expressed differentially following BD treatment actually, including shorter term response and longer term response. The results revealed that there are more down-regulated genes than up-regulated genes after BD treatment.

### 2.4. Gene Ontology Enrichment Analysis of Eight Clusters

The GOenrichment analysis of the genes in each cluster shown in Figure 5 identified the biological processes, molecular functions and cellular processes that characterize each cluster using BLAST2GO (Table 2). Although genes of eight clusters are shown in the table, cluster I and cluster V are more significant for showing DEGs functions as they contained BD-induced or -repressed genes at all three time points.

This analysis revealed that the transcripts in Cluster I, which were up-regulated by BD treatment in all the samples including 1DPT, 4DPT and 7DPT, were mainly involved in regular biological processes, such as translation, DNA replication, the oxidation-reduction process, and the signal transduction and metabolic process. Transcripts down-regulated by BD in all treated samples in Cluster V were involved in pathogenesis, chlorophyll biosynthetic process, protein import into peroxisome matrix, nitrogen compound metabolic process, and FtsZ-dependent cytokinesis. In addition, Cluster V also showed a significant representation of transcripts related to the pheromone activity, calcium channel activity, photosystem II oxygen evolving complex, and hydrolase activity.

### 2.5. Metabolic Pathway Analysis

DEGs in each cluster were enriched in different KEGG pathways (Table 3). Genes that showed an increase in transcript abundance in response to BD in Clusters I to IV were associated with ribosome, and plant hormone signal transduction. Moreover, up-regulated genes were also associated with glyoxylate and dicarboxylate metabolism, plant secondary metabolites (thiamine, amino sugar, nucleotide sugar, and alpha-linolenic acid), terpenoid biosynthesis (monoterpenoid, sesquiterpenoid and triterpenoid), chlorophyll metabolism (porphyrin and carotenoid), phenylalanine metabolism, and pyruvate metabolism. Conversely, genes down-regulated by BD treatment in Clusters V to VIII were associated with protein processing in the endoplasmic reticulum, spliceosome, secondary cell wall structure (cutin, suberine and wax biosynthesis), metabolism of xenobiotics by cytochrome P450, the mitogen-activated protein kinase (MAPK) signaling pathway, arachidonic acid metabolism. In addition, genes enriched in glutathione metabolism, plant–pathogen interaction and photosynthesis pathways were also down regulated. Also consistent with the GO enrichment result is that BD suppressed the expression of plant–pathogen interaction genes and photosynthesis pathway genes.

### 2.6. BD Regulated Transcription Factors

**Figure 6:**
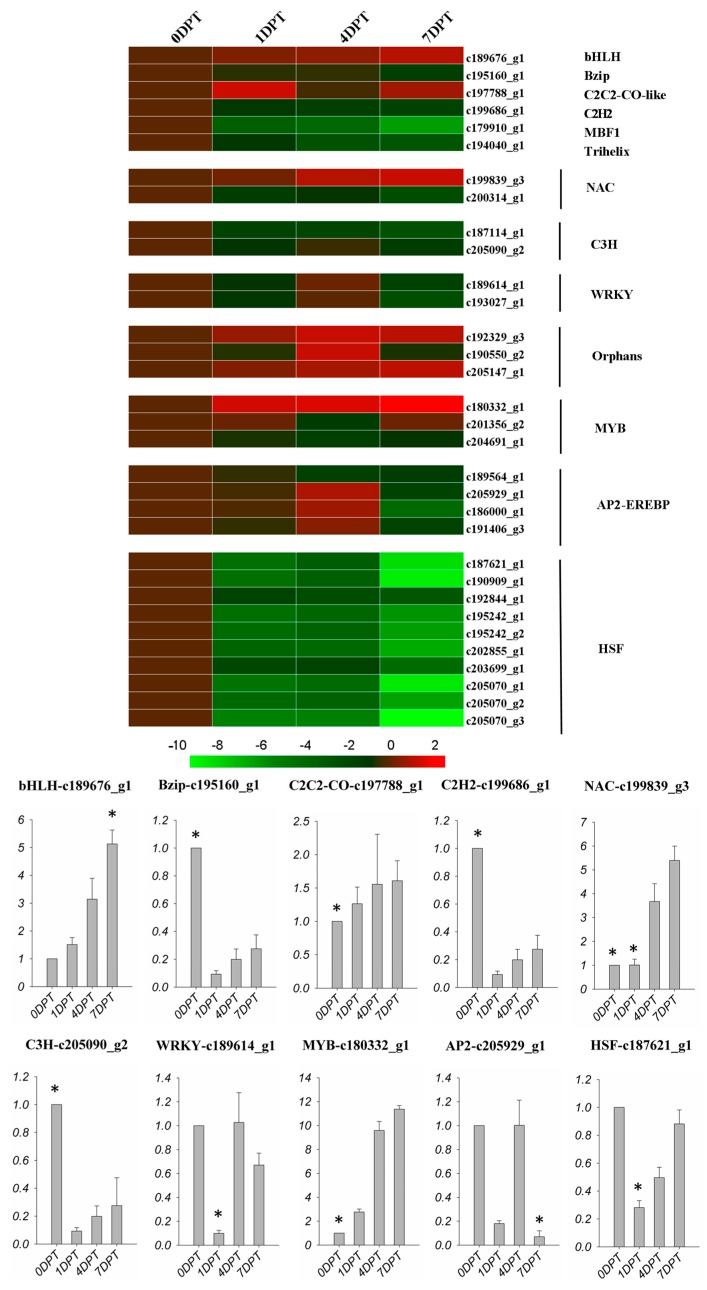
Expression profiles of transcription factors in DEGS following BD treatment. Colors indicate the log_2_(foldchange) of expression values scaled from 2 to −10. Red indicates increased expression and green indicates decreased expression relative to the 0DPT. The qPCR data was exhibited by fold change relative to the 0DPT, which was presented as 1. The asterisk on the bar presented significantly higher or lower than the other bars (*p <* 0.01).

In this study, iTAK software was used to determine the TF families related with BD responses. Figure 6 showed the expression level at each time point of the transcription factors responding to BD treatment. BHLH, C2C2-CO-like, one NAC factor and one v-myb avian myeloblastosis viral oncogene homolog (MYB) factor showed an up-regulated trend, while basic leucine zipper (Bzip), C_2_H_2_, MBF1, Trihelix, C3H, WRKY, one NAC, two MYB, one AP2-EREBP, and all of the heat shock factors (HSFs) were down-regulated. Furthermore, three of the AP2-EREBP TFs were up-regulated first, then down-regulated. Three TFs were defined as orphans, meaning TFs with undefined functions. Among 10 selected genes examined by quantitative real time polymerase chain reaction (qRT-PCR), nine of them exhibited the same pattern of responses to BD treatment as found in the RNA-seq analysis (Figure 6).

### 2.7. BD-Regulated R-Gene of Creeping Bentgrass

**Figure 7:**
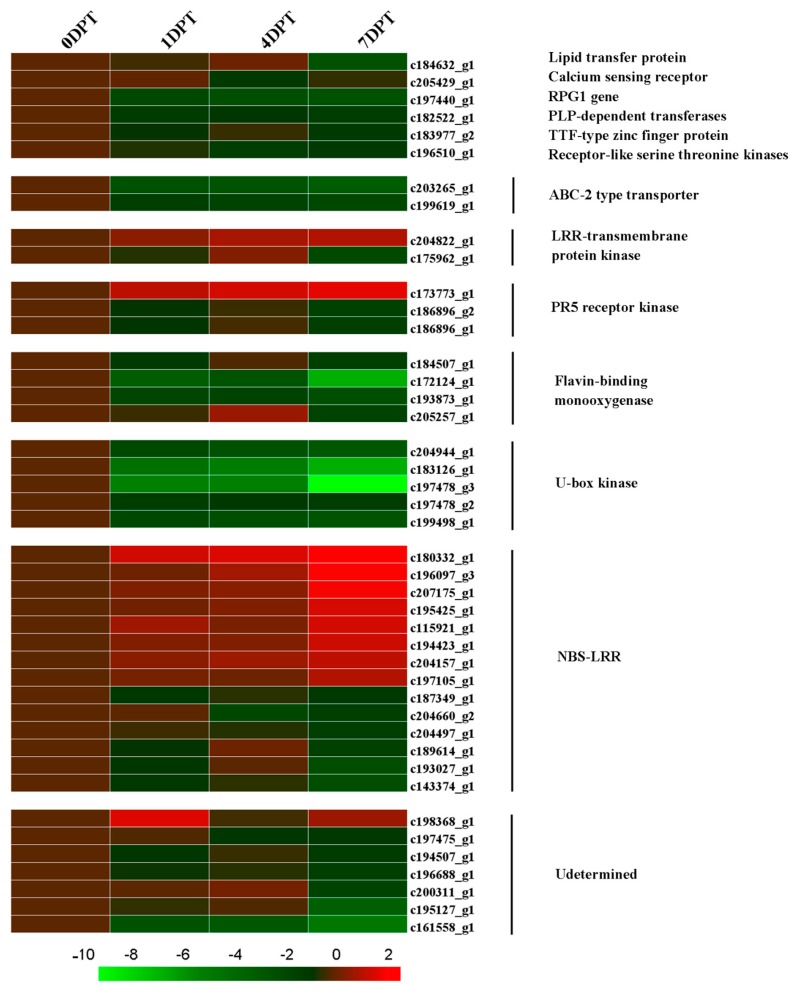

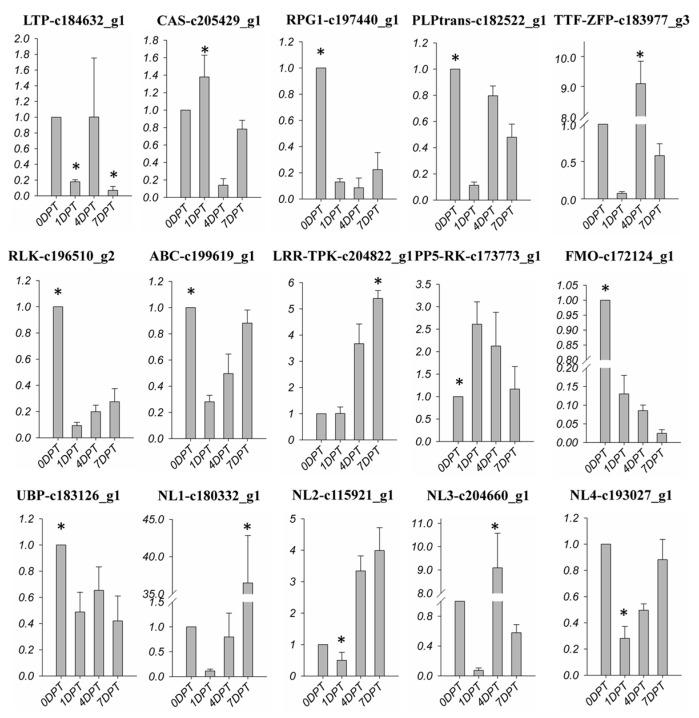
Expression profiles of *R*-gene in DEGS following BD treatment. Colors indicate the log_2_(foldchange) of expression values scaled from 2 to −10. Red indicates increased expression and green indicates decreased expression relative to the 0DPT. The qPCR data was exhibited by fold change relative to the 0DPT, which was presented as 1. The asterisk on the bar presented significantly higher or lower bars (*p < 0.01*).

All of the 504 DEGs were blasted with the all protein database of plant resistance gene database (PRGDB); 54 genes were functionally annotated with the 16 plant *R*-gene family. Among these genes, seven were homologous with heat shock cognate protein 70 (HSP), which was consistent with the down-regulated trend of HSFs. Two of the annotated genes were determined to be AP2 domain class transcription factors, and another two were found to be NAC domain containing proteins, this was also confirmed by TF analysis. Some other PRGs were also blasted with the DEGs (Figure 7), including lipid transfer protein, calcium sensing receptor, *RPG1* gene, PLP-dependent transferases, receptor-like serine threonine kinase, ABC-2 type transporter, flavin-binding monooxygenase and U-box kinase, which were down-regulated by BD treatment. Furthermore, one LRR-transmembrane protein kinase, one PR5 receptor kinase, and 8 *NBS-LRR* genes were up-regulated in response to BD. In addition, seven of the PRGs had undetermined functions. A total of 15 genes were used to confirm the expression alteration of bioinformatics analysis. At least one gene was chosen in each family for qRT-PCR. The fold change of the qRT-PCR results had the same trend as the RNA-seq analysis of each chosen gene (Figure 7).

### 2.8. BD-Induced DEGs Related with Plant Biotic Stress

**Figure 8:**
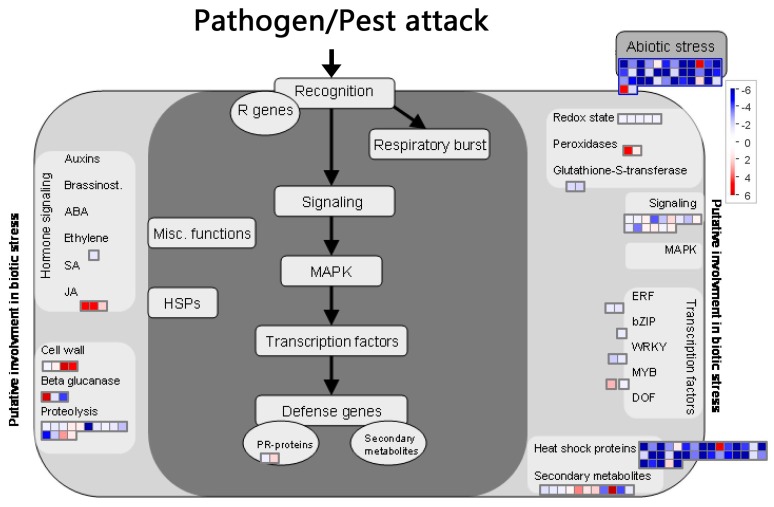
Mapman software visualization of genes related to biological stress. Genes differentially expressed (|log_2_(foldchange)| > 1, *q*-value < 0.005) after BD treatment on creeping bentgrass leaves. Red color represents up-regulated genes and blue color represents down-regulated genes. The dark gray rectangle includes genes directly involved in biotic stress responses and the light gray rectangle includes genes potentially involved in biotic stress response. ABA, abscisicacid; bZIP, basic region leucine zipper; DOF, DNA-binding with one finger; ERF, ethylene responsive factor; HSP, heat shock protein; JA, jasmonicacid; MAPK, mitogen activated protein kinase; MYB, myeloblastosis; PR, pathogenesis-related; R, resistance; SA, salicylicacid.

DEG function analysis with Mapman software (Figure 8) revealed that the TFs ERF, Bzip, and WRKY were significantly down-regulated, one MYB TF was up-regulated, and most of the HSP were down-regulated. Furthermore, several NBS-LRR genes, protein kinase genes, and LRR-transmembrane protein kinases were up-regulated. These results were consistent with the TFs and *R*-gene analysis. Genes mapped with secondary metabolites (terpene synthase, transferase protein, aromatic compounds synthase and dehydrogenase family), peroxidase, beta glucanase (hydrolase activity), and cell wall (glycosyl hydrolase) were up-regulated, which was also found with GO enrichment. One gene related to xyloglucan endotransglycosylase (XET) in cell wall clusters was up-regulated by more than five folds, which was also found in the GO enrichment analysis. Three genes mapped with JA-related clusters (lipoxygenase, allene oxide synthase) were significantly up-regulated.

## 3. Discussion

The results from the disease evaluation and direct antifungal activity tests suggested that 250 µM BD was most effective in suppressing disease damage by *R. solani* in creeping bentgrass. This was not due to the control of fungal growth, but because of the induced disease resistance of plants. The induced resistance of creeping bentgrass to *R. solani* could be associated with the transcriptomic changes activated by BD, as discussed below.

Transcriptome sequencing analysis identified 504 *BD-responsive* genes, which were classified into eight functional clusters (Figure 5). There were more down-regulated genes than up-regulated genes after BD treatment. This result is similar to the research of Hao et al. [32], who also found the transcriptomes of BVC-treated *Arabidopsis* leaves showed more down-regulated genes than up-regulated genes. This might be explained by the research of Cartieaux et al. [33], who found that after *Arabidopsis* was treated with PGPR, nearly all of the DEGs related with defense response, metabolism and signal transduction were down-regulated. However, when the plant was then inoculated with the pathogen, all of these genes were significantly up-regulated. Thus we can assume that after the BD-treated creeping bentgrass was inoculated with the pathogen, the DEGs might have changed their expression trend.

Genes that showed an increase in transcript abundance in response to BD in Clusters I to IV were associated with the ribosome, and plant hormone signal transduction, which were also up-regulated in *Arabidopsis* treated with BVC released from PGPR [32]. Moreover, up-regulated genes were also associated with glyoxylate and dicarboxylate metabolism, plant secondary metabolites (thiamine, amino sugar, nucleotide sugar, and alpha-linolenic acid), terpenoid biosynthesis (monoterpenoid, sesquiterpenoid and triterpenoid), chlorophyll metabolism (porphyrin and carotenoid), phenylalanine metabolism, and pyruvate metabolism. Howell et al. reported [34] that *Trichoderma virens* treatment induced terpenoid synthesis of cotton and it may be an important mechanism against *R. solani*. l-phenylalanine ammonia-lyase (PAL+) is an important enzyme in the phenylalanine metabolism pathway; Shadle et al. [35] found that tobacco plants over-expressing PAL+ exhibited markedly reduced susceptibility to fungal infection. This result suggested that BD might induce plant resistance to disease through the induction of disease–defense related secondary metabolites, such as terpenoid and phenylalanine.

The down-regulated transcripts in Cluster V included those involved in pathogenesis, chlorophyll biosynthetic process, protein import into the peroxisome matrix, the nitrogen compound metabolic process, and FtsZ-dependent cytokinesis. BD down-regulated transcripts also included those related to pheromone activity, calcium channel activity, the photosystem II oxygen evolving complex, and hydrolase activity. Previous studies have reported that BVCs resulted in the up-regulation of photosynthesis genes [36]; However, in our study the genes related to photosystem II (chlorophyll biosynthetic process, nitrogen compound metabolic process, photosystem II oxygen evolving complex, magnesium chelatase complex) were down regulated. In plant–pathogen interactions, calcium is known to have an important role and to be significantly induced in the regulation of early events after elicitor perception [37]. In this study, calcium channel activity-related genes were down-regulated in all treated samples. Based on these changes, we can come up with a conjecture that without pathogen inoculation, BD could suppress the expression of some of the regular stress-related genes of creeping bentgrass. This conjecture can also be verified by the GO enrichment of other clusters of down-regulated genes. Chitinase is a key enzyme for plants against fungal disease [38], while genes related with chitinase activity were down-regulated by BD treatment. Glycerolipid metabolic-process-related genes were also suppressed by BD treatment, and glycerolipid metabolic could regulate oleic acid levels, which could lead to the modulation of SA- and JA-mediated defense gene expression [39]. The down-regulation of glycerolipid metabolic process can also lead to the down-regulation of pathogenesis-related genes. Moreover, the down-regulated genes included autophagy and apoptotic process-related genes, which were typical up-regulated genes of plants after disease attack. Some genes that were down-regulated by BD treatment in Clusters V to VIII were associated with protein processing in the endoplasmic reticulum, spliceosome, secondary cell wall structure (cutin, suberine and wax biosynthesis), metabolism of xenobiotics by cytochrome P450, MAPK signaling pathway, and arachidonic acid metabolism. In addition, genes in glutathione metabolism, plant–pathogen interactions and photosynthesis pathways were also down-regulated, which is consistent with transcriptomic studies reporting the interaction between plants and PGPR [40,41,42].

Transcriptional factors, including bHLH, Bzip, C_2_H_2_, NAC, C3H, WRKY, MYB, AP2-EREBP, and HSF are broadly involved in the signal transduction of plant tolerance and stress defense. The bHLH (basic helix-loop-helix) family plays an essential and often conserved role in the plant kingdom, which is involved in JA-modulated processes in *Arabidopsis* [43]. MYC2 and some other homologue transcription factors belong to the bHLH IIIe subclade, which positively contributes to the general JA response [43]. MYC2 is involved in the majority of JA-related processes. MYC2 is also an important transcriptional regulator of priming during ISR [44]. The up-regulation of bHLH TFs suggested that BD may elicit a JA-mediated stress defense. Group D genes of the bZIP family participate in plant defense against pathogens. BZIP/TGA factors interact with the NPR protein, which is necessary for *PR* gene induction [45]. Also, the bZIP factors might be involved in integrating the systemic signals induced by salicylic acid (SA) at the PR promoter level [45]. In this study, the down-regulation of bZIP TFs by BD indicated that the induced disease resistance was not related to SA pathways. C2C2-CO-like, C2H2 and C3H were the main *zinc finger TFs* genes. C2H2 were identified to be regulated in the PGPR-induced plant transcriptome [33]. *Zinc finger TFs* genes were also reported in response to environmental stress in *Arabidopsis* [46]. However, only one C2C2-CO-like TF was up-regulated in the present study, C2H2 and C3H TFs were down-regulated. NAC proteins are a family of plant-specific transcription factors with diverse biological functions [47]. One report has demonstrated that overexpression two proteins of the NAC family can regulate JA-induced expression of defense genes [48]. Another report has demonstrated that the *Arabidopsis* NAC protein ATAF2 functioned as a repressor of *PR* genes [49]. This is interesting because one of the NAC protein genes was up-regulated in the creeping bentgrass transcriptome after being treated with the synthetic PGPR volatile compound. This could be related to plant ISR induction. However, another NAC gene was down-regulated, which might be explained by the repressive function of part of the NAC family. WRKY factors are crucial regulators of the defense transcriptome and disease resistance, and some of the WRKY factors are required for both basal defense and *R*-gene mediated disease resistance [50], as well as Ap2-EREBP (APETALA 2/ethylene responsive element binding protein) TFs. Two WRKY factors and four Ap2-EREBP were up-regulated first then down-regulated during short-term BD treatment in this study. This indicated that WRKY and Ap2-EREBP are short-term response regulators of plant resistance induced by BD. It can be assumed that these genes could be up-regulated again when the pathogen attacks, the way that the defense response genes of PGPR-treated *Arabidopsis* up-regulated again after *Pseudomonas syringae* was inoculated with the plant [33]. MYB recognition elements were represented from the parsley disease resistance related genes (phenylalanine ammonia-lyase (PAL) and 4-coumarate: *CoA ligase* (*4CL*) genes) [51]. This previous research suggested that one type of MYB TF was positive related with plant defense. Two MYB TFs were up-regulated in this study, which is consistent with Logemann’s research. Heat shock factors (HSF) are typically induced or up-regulated in response to biotic or abiotic stresses [52]. In this study, HSF transcripts showed significant down-regulation by BD treatments, which may reflect the reduced susceptibility of plants to pathogens due to BD effects. In Cartieaux’s study, the HSP 70 family of PGPR-treated *Arabidopsis* was also down-regulated, while it was found to be up-regulated immediately after pathogen attack [33].

In searching the plant resistance gene database (PRGDB) among 504 genes, 54 genes were functionally annotated with the 16 plant *R*-gene family. Among these genes, seven were homologous with heat shock cognate protein 70 (HSP), which was consistent with the down-regulated trend of HSF. Two of the annotated genes were determined to be an AP2 domain class transcription factor, and another two were found to be NAC domain containing proteins, this was also confirmed by TF analysis. Lipid transfer protein (LTP) is a classic pathogenesis-related (PR) protein, which was defined as PR-14, expressed by lipid transfer protein genes [53]. Plant SAR induction is usually related with plant *PR* gene induction [54]. The decreased expression of *LTP* genes in this study indicated that the signaling pathways of BD-induced disease resistance are different from SAR. Calcium sensing receptors, which are also called CAS, are a protein localized in the chloroplast thylakoid membranes in *Arabidopsis*; it has been suggested that these are activated by Ca^2+^ [55]. They were reportedly involved in the pathogen-associated molecular patterns (PAMP)-induced expression of defense genes [56]. PAMP signals evoke a CAS-dependent transient Ca^2+^ signal in chloroplasts. In the present study, the down-regulation of the calcium sensing receptor could be explained by the fact that the plant was without pathogen inoculation. This can also explain the down-regulation of the RPG1 homologue, a rust-resistance gene first released in barley, which is a homologue with receptor kinases [57], as well as the receptor serine threonine kinases (RSTK) similar gene, which plays a central role in signaling during pathogen recognition [58]. *Flavin-dependent monooxygenase (FMO)* genes were reported to be critical for the development of SAR, as well as for the accumulation of salicylic acid and the expression of defense-related genes [59]. Four *FMO* genes were significantly repressed by the BD-treated creeping bentgrass plant; while the plant still showed resistance to *R. solani*, it demonstrated that the disease-resistance signaling pathway is far different from SAR. One of the *PR-5* genes, which is called the thaumatin-like protein gene, was induced by BD treatment, but the other two were repressed. *PR-5* was reported can be induced by SA, while it can be induced to a greater extent by the synergy of ethylene and methyl jasmonate [60]. Based on this previous research, the induction of *PR-5* genes by BD might affect creeping bentgrass resistance against disease. BD is not an as strongly induced function as ethylene and methyl jasmonate, so just one gene of *PR-5* was induced, while the other two were repressed, which also explains that BD induction is not dependent on SAR pathway. The U-box domain-containing protein kinase family members were down-regulated in this study. Proteins contained U-box domains were reported as negative regulators of plant cell death and defense [61]. Therefore, down-regulation is helpful to plant defense induction. Nucleotide binding site (NBS) leucine rich repeats (LRR) are well known plant *R*-gene analogs (RGAs) [62]. In the current study, numbers of DEGs were classified as NBS-LRR type *R*-genes, and more than half of them were up-regulated after being treated with BD. NBS-LRR proteins are involved in the recognition of specialized pathogen effectors that are thought to provide a virulence function in the absence of the cognate resistance (*R*) gene [63]. The up-regulated *NBS-LRR-related* genes can prepare for the recognition of pathogen effectors. This might be the significant cause of creeping-bentgrass-induced resistance to *R. solani*. There were still some *NBS-LRR* genes down-regulated, and the variation of their expression can be further determined by transcriptome analysis when the BD-treated plant is inoculated with the pathogen, which is also the next step of our research.

To determine the impact of BD on plant-induced resistance, the analysis focused on genes potentially involved in biotic stress. Identification of these genes was improved using Mapman annotation software. This additional analysis, illustrated in Figure 8, confirms observations made with TFs and *R*-gene analysis. Notably, one gene related with xyloglucan endotransglycosylase (XET) in cell wall clusters was up-regulated by more than five times, this was consistent with the GO enrichment analysis. XET belongs to a class of cell wall enzymes that can enable microfibril relaxation and cell expansion, and are thus related with the plant growth rate [64] Although we did not observe the plant growth effect of BD, this analysis was supported by the report that BD and BVC can promote growth in *Arabidopsis* [14]. Three genes mapped with a JA-related cluster (lipoxygenase, allene oxide synthase) were also significantly up-regulated. Lipoxygenase and allene oxide synthase were key enzymes in the JA biosynthesis pathway, which are important signal molecules involved in induced resistance to pathogen infection [65,66]. Their up-regulation indicated the higher expression of JA, which might explain the induced disease resistance of creeping bentgrass.

## 4. Materials and Methods

### 4.1. Plant Material and Treatment

Creeping bentgrass (cultivar Penn A4) seeds were kindly provided by Clover Group (Beijing, China). The seeds were soaked in distilled water for 5 h before they were surface sterilized by rinsing in 70% ethanol for 30 s, followed by treatment with 10% sodium hypochlorite solution for 20 min. Sterilized seeds (0.25 g) were placed evenly within a jam jar (6 cm diameter, 8.5 cm height, 240 cm^3^ volume) filled with 100 g of dry sandy loam soil and 20 mL autoclaved tap water. Jars were tightly sealed with translucent lids, and incubated at 25 °C in a culture room under fluorescent lights with photosynthetically active radiation (PAR) of 500 µmol m^−2^ s^−1^ for 15 days.

Following the 15-day growing period, 10 mL BD (Xiya Reagent, Chengdu, China) at 150, 250, 350, 450, or 550 µM was added to the soil medium in the jars. Control plants were treated with distilled water. The control and each concentration had three replicates.

### 4.2. Inoculation and Disease Assessments

*R. solani* was grown on potato dextrose agar medium (PDA) and incubated at 25 °C for seven days. One PDA plate with fungus grown on it was cut into pieces and added into 500 g of autoclaved moist wheat seeds in jam jars. The mixture of seeds and *R. solani*-infected PDA was incubated for two weeks at 25 °C. After the wheat seeds were thoroughly colonized, they were transferred to large trays to allow to dry for up to one week. The dry wheat seeds were ground into powder for inoculating. The inoculum was stored at 4 °C under low relative humidity conditions.

Plants of creeping bentgrass treated with different concentration of BD and water were incubated with 0.01 g of inoculum in each jar for seven days. The jars were incubated in a culture room at 25 °C and 500 µmol m^−2^ s^−1^ PAR. Disease rating was taken at four, seven, 10, and 15 days post inoculation (DPI) to estimate the disease level in terms of mycelial growth and the yellowing of turfgrass leaves.

### 4.3. Antimicrobial Activity Test

*R. solani*, 5 mm min diameter, was grown on PDA medium and transferred to PDA amended with 0, 50, 500, 1000, or 5000 µM BD and sealed with parafilm. Four replicate plates per treatment were prepared. Plates were incubated at 28 °C for up to nine days, and the radial growth of the fungus was measured daily.

### 4.4. Sample Preparation and RNA Extraction

The disease infection test showed that 250 µM BD was most effective in suppressing disease symptoms. Therefore, leaf samples collected before and at one, four, and seven days post 250 µM BD treatment (days post treatment, DPT) were used for the transcriptomic analysis. Leaf samples were immediately frozen in liquid nitrogen for RNA isolation. The total RNA of creeping bentgrass leaves was isolated using the Plant RNA Kit (OMEGA, Norcross, GA, USA) according to the manufacturer’s instructions. RNA degradation and contamination were monitored on 1% agarose gels and the purity was checked using the NanoPhotometer^®^ spectrophotometer (IMPLEN, Los Angeles, CA, USA). The RNA integrity number (RIN) was assessed using the RNA Nano 6000 Assay Kit of the Agilent Bioanalyzer 2100 system (Agilent Technologies, Santa Clara, CA, USA). High quality total RNA samples were selected for further processing.

### 4.5. Library Preparation and Transcriptome Sequencing

To comprehensively survey the genes associated with disease resistance induced by BD, total RNAs were extracted from creeping bentgrass cv. Penn A4 sprayed with the chemical for 0, 1, 4 and 7 days. Raw Illumina sequences were deposited in the National Center for Biotechnology Information (NCBI) and can be accessed in the sequence read archive (SRA) database (https://www.ncbi.nlm.nih.gov/sra) under accession SRR5763094-SRR5763097 for DPT0, DPT1, DPT4, DPT7, respectively.

RNA samples were utilized to make the cDNA libraries, each library being generated from an equivalent mixture of three independent RNA preparations. Sequencing libraries were generated using NEBNext^®^ Ultra™ RNA Library Prep Kit for Illumina^®^ (NEB, Ipswich, MA, USA) following the manufacturer’s recommendations. Index codes were added to attribute sequences to each sample. Briefly, mRNA was purified from total RNA using poly-Toligo-attached magnetic beads. Fragmentation was carried out using divalent cations under elevated temperatures in NEBNext first strand synthesis reaction buffer (5X). First strand cDNA was synthesized using a random hexamer primer and M-MuLV reverse transcriptase (RNase H-). Second strand cDNA synthesis was subsequently performed using DNA polymerase I and RNase H. Remaining overhangs were converted into blunt ends via exonuclease/polymerase activities. After adenylation of 3′ ends of DNA fragments, NEBNext adaptors with a hairpin loop structure were ligated to prepare for hybridization. In order to select cDNA fragments of preferentially 150~200 bp in length, the library fragments were purified with the AMPure XP system (Beckman Coulter, Beverly, MA, USA). Then 3 μL USER enzyme (NEB, USA) was used with size-selected, adaptor-ligated cDNA at 37 °C for 15 min followed by 5 min at 95 °C before PCR. Then PCR was performed with Phusion High-Fidelity DNA polymerase, universal PCR primers and index (X) primer. At last, PCR products were purified (AMPure XP system) and library quality was assessed on the Agilent Bioanalyzer 2100 system. The clustering of the index-coded samples was performed on a cBot cluster generation system using TruSeq PE cluster kit v3-cBot-HS (Illumina) according to the manufacturer’s instructions. After cluster generation, the Illumina Hiseq 2000 platform was used for deep sequencing of the four cDNA libraries from both 5′- and 3′- end by 150-bp reads at Novogene Bioinformatics Institute (Beijing, China).

### 4.6. Transcriptome Assembly and Annotation

To ensure the reliability of the libraries, clean reads were obtained by removing reads containing adapter, reads containing ploy-N, and low-quality reads from the raw data. Due to the absence of reference genomic sequences, de novo assembly was applied to construct transcripts from these RNA-seq reads. Trinity (v2014-04-13) [67], which has been shown to perform the best for restoring full-length transcripts, as described previously, was used for transcriptome assembly with min_kmer_cov set to 2 by default and all other parameters set to default. The sequences with >95% similarity were grouped into one class, and the longest sequence of each class was treated as a unigene during later processing. This transcriptome unigene assembly project has been deposited at GenBank under the accession GFQK00000000. The version described in this paper is the first version, GFQK01000000.

Taxonomic and functional annotation of the transcripts was conducted using Blast+(V2.2.28+) for the annotated with nr (nonredundant protein sequences), and the nt (nonredundant nucleotide sequences) database from the National Center for Biotechnology Information (NCBI, National Institutes of Health, Bethesda, MD, USA) and Swiss-Prot (A manually annotated and reviewed protein sequence database), KAAS for annotation with KEGG (Kyoto Encyclopedia of Genes and Genomes), HMMER3 for Pfam (a large collection of protein families) and Blast2go for GO (gene ontology) annotation.

### 4.7. Transcript Expression Analysis

The full-length reads were directly mapped to the reference unigene using RSEM (v 1.2.15) alignment [68]. Mapping parameters allowed for only uniquely mapped reads without mismatches. The read counts of the transcripts with a reciprocal match to the reference transcriptome were counted and extracted for gene expression analysis. To identify differential expression genes (DEGs) in creeping bentgrass treated with BD, differential gene expression analysis was performed between any two samples among four libraries using the DEGseq (2010) R package. Genes with |log_2_(foldchange)|>1 (*q*-value < 0.005) [30] were designated as differentially expressed genes(DEGs) [31]. To compare these results with the transcriptome background, all DEGs were subjected to GO enrichment analysis using the GOseq R package [69]. KEGG pathway enrichment analysis used the KOBAS [70] software to test the statistical enrichment of DEGs in KEGG pathways.

iTAK [71] (http://bioinfo.bti.cornell.edu/tool/itak) was used to determine the transcription factors families related with BD treatment response. All the DEGs were blasted with the plant resistance gene database (PRGDB) to find resistance genes (*R*-gene) in the BD response genes of creeping bantgrass. PRGDB is an open and daily updated space regarding plant *R*-genes, in which all information about this family is stored, curated and discussed [72]. Mapman was used to generate the biotic stress-related map, which could classify the DEGs with different response clusters to biotic stress.

Total RNA was isolated from leaf tissue of 0, 1, 4 and 7-day BD-treated grass, while leaves harvested from water-treated grass were used as a control. A total of 25 transcripts were selected for verifying the RNA-seq analysis. qRT-PCR reactions were conducted in three independent technical repeats with four biological replicates. The primer design can be found in the supplementary material Table S1. The qRT-PCR data was analyzed using the ΔΔCT method described by Livak and Schmittgen [73].

### 4.8. Statistical Analysis of Data

The expression levels of transcripts for control and BD-treated cDNA pools were statistically analyzed by *t*-Test using SPSS software (version 20.0, SPSS Inc., Chicago, IL, USA) and means of difference between treatments were considered statistically significant when *p* < 0.01.

## 5. Conclusions

In summary, synthetic BD induced plant defense against *R. solani* for creeping bentgrass. Transcriptomic analysis demonstrated that BD suppressed the expression of some regular stress-related genes in creeping bentgrass, such as pheromone activity, calcium channel activity, photosystem II oxygen evolving complex, and hydrolase activity, while it up-regulated defense-related TFs, such as bHLH, C2C2-CO and NAC. Other genes related to disease resistance, such as JA signaling, LRR-transmembrane protein kinase, PR5 receptor kinase, and NBS-LRR domain containing *R*-genes were also significantly up-regulated. These results suggest that BD may induce the disease resistance of plants involving changes to the transcriptome in induced systemic resistance pathways. Further research is required to confirm the direct relationship of BD-responsive genes with disease resistance in pathogen-inoculated plants.

## Figures and Tables

**Table 1:** Summary of assembled transcriptome and function annotation of creeping bentgrass.

	Items	Number of bp/Reads/Transcripts
Sequencing and assembly	Total raw reads	258,606,402
	Total clean reads	252,117,292
	Total transcripts	466,761
	Total length of transcripts (bp)	324,797,015
	Transcripts with N50 length (bp)	1100
	Transcript mean length (bp)	696
	Total unigenes	334,212
	Total length of unigenes (bp)	191,363,312
	Unigenes with N50 length (bp)	791
	Unigenes mean length (bp)	573
Function annotation	NR database	130,367
	NT database	72,777
	KO database	41,963

**Table 2:** GO enrichment analysis of DEGs in eight clusters (*q* < 0.05).

Cluster	Biological Processes	Molecular Functions	Cellular Component
I	Translation	Catalytic activity	Intracellular
	Phosphorelay signal transduction system	RNA binding	Transcription factor
	Metabolic process	Enzyme regulator activity	Membrane
	DNA replication		Respiratory chain complex
	Oxidation-reduction process		
II	Lipopolysaccharide core region biosynthetic	Metal ion binding	Transcription factor complex
	Response to oxidative stress	Hydrolase activity	Component of membrane
	Phospholipid catabolic process	Fructose-bisphosphate aldolase activity	
		Diacylglycerol kinase activity	
III	Thiamine biosynthetic process	Transcription factor activity	Small-subunit processome
	Proteolysis	Ubiquitin binding	Peroxidase activity
	RNA modification		
IV	Unsaturated fatty acid biosynthetic process	Terpene synthase activity	Fatty acid synthase complex
	Ferrous iron transport	Serine-type endopeptidase inhibitor activity	Chloroplast
	Response to biotic stimulus	Malate synthase activity	Cell wall
	Cellular aromatic compound metabolic	Xyloglucosyl transferase activity	
		Peroxidase activity	
V	Pathogenesis	Calcium channel activity	Photosystem II oxygen evolving complex
	Chlorophyll biosynthetic process	Pheromone activity	Magnesium chelatase complex
	Protein import into peroxisome matrix		
	Nitrogen compound metabolic process		
	FtsZ-dependent cytokinesis		
VI	Starch metabolic process	Chitinase activity	Cell junction
	Sucrose metabolic process	Calmodulin binding	Cell wall
	Response to stress	ATPase activator activity	
	Glycerolipid metabolic process		
	Chitin catabolic process		
VII	Regulation of transcription, DNA-templated	Adenosylcobinamide kinase activity	U1 snRNP
	Pantothenate biosynthetic process	Aldehyde-lyase activity	Holliday junction helicase complex
	Autophagy		
	Cobalamin biosynthetic process		
	Apoptotic process		
VIII	Cell adhesion	Enzyme inhibitor activity	Plasma membrane
	Photorespiration	Flavin adenine dinucleotide binding	
	Oligosaccharide biosynthetic process	Nutrient reservoir activity	
	Galactose metabolic process		
	Benzoate metabolic process		
	Pectin catabolic process		

**Table 3:** KEGG pathway enrichment analysis of differentially expressed transcripts in eight clusters (*q* < 0.05).

Cluster	KEGG Pathway	*q*-Value
I	Ribosome	4.59 × 10^−36^
	Plant hormone signal transduction	0.0251
	Glyoxylate and dicarboxylate metabolism	0.0244
II	Porphyrin and chlorophyll metabolism	0.0100
	Nitrogen metabolism	0.0100
	Fructose and mannose metabolism	0.0100
	Phenylalanine metabolism	0.0100
III	Thiamine metabolism	0.0112
	Circadian rhythm-plant	0.0173
	Amino sugar and nucleotide sugar metabolism	0.0251
	Carbon metabolism	0.0453
IV	Ribosome biogenesis in eukaryotes	0.0181
	Monoterpenoid biosynthesis	0.0183
	alpha-Linolenic acid metabolism	0.0181
	Pyruvate metabolism	0.0181
	Sesquiterpenoid and triterpenoid biosynthesis	0.0301
	Carotenoid biosynthesis	0.0357
V	Protein processing in endoplasmic reticulum	1.23 × 10^−17^
	Spliceosome	0.0125
	Cutin, suberine and wax biosynthesis	0.0485
	Metabolism of xenobiotics by cytochrome P450	0.0157
	MAPK signaling pathway	0.0385
	Arachidonic acid metabolism	0.0459
	Phenylalanine metabolism	0.0732
VI	NOD-like receptor signaling pathway	8.38 × 10^−6^
	Glutathione metabolism	0.0304
	Ubiquinone and other terpenoid-quinone biosynthesis	0.0304
	Phenylpropanoid biosynthesis	0.0340
	Cyanoamino acid metabolism	0.0484
VII	Photosynthesis - antenna proteins	5.97 × 10^−7^
	NOD-like receptor signaling pathway	6.25 × 10^−5^
	Carbon fixation in photosynthetic organisms	0.0061
VIII	NOD-like receptor signaling pathway	9.69 × 10^−5^
	Plant-pathogen interaction	0.0012
	Photosynthesis	0.0301
	Brassinosteroid biosynthesis	0.0185

## Supplementary Materials

Supplementary Materials are available online.
